# Learning about COVID-19: a qualitative interview study of Australians’ use of information sources

**DOI:** 10.1186/s12889-021-10743-7

**Published:** 2021-04-07

**Authors:** Deborah Lupton, Sophie Lewis

**Affiliations:** 1 Vitalities Lab, Centre for Social Research in Health and Social Policy Research Centre, UNSW Sydney, Sydney, Australia; 2 Centre for Social Research in Health, UNSW Sydney, Sydney, Australia

## Abstract

**Background:**

A multitude of information sources are available to publics when novel infectious diseases first emerge. In this paper, we adopt a qualitative approach to investigate how Australians learnt about the novel coronavirus and COVID-19 and what sources of information they had found most useful and valuable during the early months of the pandemic.

**Methods:**

In-depth semi-structured telephone interviews were conducted with a diverse group of 40 Australian adults in mid-2020 about their experiences of the COVID-19 crisis. Participants were recruited through Facebook advertising. Detailed case studies were created for each participant, providing the basis of a thematic analysis which focused on the participants’ responses to the questions about COVID-19-related information sources.

**Results:**

Diverse sources of COVID-19-related information, including traditional media, online media and in-person interactions, were actively accessed, appraised and engaged with by participants. There was a high level of interest in COVID-19 information as people grappled with uncertainty, anxiety and feeling overwhelmed. Certain key events or experiences made people become aware that the outbreak was threatening Australia and potentially themselves. Most people demonstrated keen awareness that misinformation was rife in news outlets and social media sites and that they were taking steps to determine the accuracy of information. High trust was placed in health experts, scientists and government sources to provide reliable information. Also important to participants were informal discussions with friends and family members who were experts or working in relevant fields, as well as engaging in-person in interactions and hearing from friends and family who lived overseas about what COVID-19 conditions were like there.

**Conclusion:**

A constantly changing news environment raises challenges for effective communication of risk and containment advice. People can become confused, distressed and overwhelmed by the plethora of information sources and fast-changing news environment. On the other hand, seeking out information can provide reassurance and comfort in response to anxiety and uncertainty. Clarity and consistency in risk messaging is important, as is responding quickly to changes in information and misinformation. Further research should seek to identify any changes in use of and trust in information sources as time goes by.

## Background

News and social media can play an important role in providing information to publics in cases of new or emerging diseases. It is vital to reach people quickly and share details in a situation in which medical and public health authorities are grappling with how the disease spreads and can best be controlled [1–5]. Major infectious disease outbreaks are highly newsworthy, typically attracting dramatic statements concerning risk [6–8]. News media coverage is often a starting point at which people start to reflect on the seriousness of pandemic risk and its implications for their own lives [2, 9]. However, news reports can also become hyperbolic or convey misinformation, leading to scepticism and lack of trust in official sources, unfounded complacency or alternatively, generating heightened feelings of fear, uncertainty, depression and anxiety [2, 10, 11].

The COVID-19 outbreak began to receive public attention in early January 2020, following reports by Wuhan health officials of a cluster of viral pneumonia cases of unknown cause affecting people in that large Chinese city in Hubei province [12]. The news and public health communication environment responding to the COVID-19 crisis has been fraught, frequently characterised by conflicting or rapidly changing information as health authorities and governments struggled to make sense of this new outbreak and identify the best way to control its spread [13–15]. COVID-19 news reporters and creators and sharers of social media content have been subjected to continual criticism for disseminating misleading or false information. The term ‘infodemic’ has been used in some popular media outlets and academic analyses to describe the wealth of ‘fake news’ and conspiracy theories circulating, particularly in online news sites and social media platforms [16–20].

Analyses of how news reporting and social media content have framed COVID-19 issues across different countries have identified marked variances. Ageism in social media content has been identified, with older people positioned as both more vulnerable and more expendable than other age groups [21]. Racism has also featured in some countries’ news reporting, particularly against Chinese people, who have been frequently positioned as to blame for the emergence of the novel coronavirus in Wuhan [22]. Politicisation and polarisation of opinion has characterised COVID-19 news in US television networks and newspapers [23], whereas strong support for government measures was evident in South Korean newspaper reporting [24]. A focus on prevention and control measures, medical treatment and research, and global or local socioeconomic influences was identified in an analysis of Chinese news articles [25].

Information provided in news coverage of COVID-19 in Australia has included reporting of the first cases and deaths and the subsequent rapid spread of the novel coronavirus around the world, accounts of statements, decisions and press conferences held by political leaders and health authorities, strategies to avoid infection, medical controversies and debates and progress towards treatments for COVID-19 and vaccines [5, 26, 27]. In Australia, very early news reporting (January 2020) focused on the ‘mystery Chinese virus’ and made continual comparisons to SARS [26]. A study of two major Australian newspapers’ COVID-19 coverage found that they were quite slow to begin covering the emerging outbreak, suggesting an initial lack of awareness that it might pose a threat to Australians. Subsequent news reporting largely focused on the social and economic impacts of the crisis. There was little blame or judgement directed at any social or national groups, although panic buyers did receive some criticism [27].

Quantitative surveys to determine how publics were responding to coverage of the COVID-19 crisis in news and social media have identified an association between COVID-19-related news and social media consumption and heightened anxiety and distress in Russia [28], China [29, 30] and USA [31]. Malaysian research found that respondents mainly used television and internet news portals to access COVID-19 information. Those who preferred government sources of information were more confident about the control of COVID-19 and believed their government was handling the crisis well [32]. Research in the UK found that people’s news consumption surged in the early months of the crisis but gradually returned to pre-crisis levels, with evidence of a growing avoidance of news. A decline in respondents’ trust in key sources of COVID-19-related news and information was also noted: particularly in relation to social media sites and government sources [33].

A comparative international online survey included nationally representative samples from Australia, New Zealand, UK, USA, Italy and South Korea [34]. For respondents in most of these countries, government and friends and family were the most trusted sources of COVID-19 information, ranked above the news media and social media. Together with New Zealanders (89%), Australians evidenced the highest levels of trust in their government (78%) to give clear and accurate advice on COVID-19. While 58% of Australians said they trusted the news media in general for COVID-19 advice, only 30% trusted information found or shared on social media more specifically.

Another online survey conducted early in the Australian nation-wide lockdown (April 2020) [35] found that Australians were consuming news media more than usual due to their interest in and concern about the pandemic. More than two-thirds said that they had been accessing news more than once a day since the outbreak. Half of the respondents were using television reporting as their main COVD news source, while 22% were accessing online news coverage and 18% news on social media. The respondents reported high levels of satisfaction with news coverage of COVID-19 (73%), but it was contributing to people’s feelings of anxiety, particularly for women and younger people. Most respondents said that they trusted health experts and scientists (85%) and to a lesser degree, government (66%) to provide information about COVID-19. Just over half said that they trusted news organisations but less than a quarter of respondents reported that they had encountered high levels of misinformation in the news or social media about COVID-19. Australians agreed that the federal government had done a good job of informing them about the pandemic (75%) and how they should respond (81%).

The surveys reviewed above are valuable in identifying trends across large populations in attitudes and practices related to COVID-19 information sources. To complement and extend such findings, qualitative social research methods provide a way of investigating people’s engagements with personal sources of information about emerging health risks such as family members and healthcare providers as well as with government sources and news and social media reporting in greater depth. This approach provides for explorations of lived experiences in sociocultural contexts [1, 2, 11, 36]. Thus far, few qualitative analyses of Australians’ responses to news and social media coverage of COVID-19 have been published. Among other issues, the ‘Australians’ Experiences of COVID-19′ study investigated people’s use and appraisal of information about COVID-19. We wanted to surface the full range of information sources upon which participants relied and those they most trusted: including but beyond media or government sources.

## Methods

The study took place during the first 6 months of the COVID-19 crisis in Australia, following the identification of the first Australian COVID-19 cases on 25 January 2020 and the implementation of a nation-wide lockdown from mid-March 2020 [37]. Forty indepth, semi-structured interviews with adults living in Australia were conducted by the second author between late May and late July 2020. In addition to the closing of international borders and some national state borders, the national lockdown included directives for people to work at home where possible, limits on household visitors, bans on public gatherings, the closing of non-essential services and schools, and physical distancing rules. The spread of COVID-19 began to be slowed by April 2020. Restrictions were progressively eased from mid-May 2020 onwards but fluctuated in response to the incidence of COVID-19 community case numbers. Restrictions were re-introduced in the state of Victoria from July until November 2020, following a significant second wave of infection in that state [37].

Due to physical distancing restrictions, the interviews for this study were conducted by telephone. This method also ensured that people living across the nation, including in regional and remote areas, had the opportunity to participate in the study. Interested potential participants responded to an advertisement about the study on Facebook. Participants were offered a gift card to compensate them for their time. Sub-quotas were set and achieved in recruitment to ensure a heterogeneous interviewee group with a spread of participants across gender, age group, and place of residence (metropolitan, regional and rural/remote areas). Facebook was chosen to advertise for recruitment because of its popularity among Australian adults. At the time this study was carried out, figures on Australian Facebook use show that 60% of all Australians (of any age) were regular Facebook users, with 50% of the Australian population logging on at least once a day [38]. Using this method of recruitment therefore proved to be fast and effective, and we easily met our sub-quotas. Table 1 shows the sociodemographic characteristics of the participants.

**Table 1:** Participant characteristics (*n* = 40)

**Gender identification**	
Female	19
Male	18
Other	3
**Age group**	
18–29	10
30–49	9
50–69	13
70+	8
**Location**	
Metropolitan	17
Regional	13
Rural/Remote	10
**Education**	
University	19
No university	21
**Ethnic/racial identification**	
Anglo-Celtic/European	33
Indigenous Australian	1
Asian	3
Central/South American	2
Middle Eastern	1

The study adopted a qualitative approach that was focused on a wide-ranging interview about the participants’ experiences of the novel coronavirus/COVID-19 during the 6 month period following identification of the first Australian cases. All interviews were audio-recorded and professionally transcribed in full. A narrative case study approach was adopted in compiling and analysing the interview materials. This approach sees the indepth interview as a form of shared storytelling, in which participants recount narratives in response to interview questions and researchers formulate their accounts into narratives [39, 40]. The second author wrote fieldnotes for each participant soon after she conducted each interview. These fieldnotes were presented in narrative form, drawing on the author’s impressions and recollections of how the participants responded to the questions. Once each interview was transcribed by a professional service and returned to the authors, both authors then used the transcripts to augment these notes, inserting illustrative direct quotations from them to configure a detailed narrative case study for each interviewee. These case studies, together with the full transcripts, comprise our research materials for analysis.

Some of our findings are reported thematically across the case studies, while in other analyses we present case studies to provide a detailed biographical narrative. For the purposes of the present paper, the set of detailed case studies formed the basis of a topical thematic analysis which focused on the participants’ responses to the questions about sources of information about COVID-19. These themes were derived as an iterative analytical process involving both authors working with the research materials of case studies we had developed together with the interview transcripts. This approach to social inquiry is directed at identifying ‘making the mundane, taken-for-granted, everyday world visible’ through interpretative and narrative practices ( [41] , p. 723). As Denzin ( [41] , p. 722) puts it, human experience (and by extension, social inquiry) ‘is a process. It is messy, open-ended, inconclusive, tangled up’. Hence our focus on interpretation and narrative as modes of analysis: the interviewees interpreted our questions in formulating their responses, and we in turn interpreted their responses in configuring the case studies, identifying themes across the cases and presenting our findings.

The first question in the interview prompted participants to think back to how and when they had first heard about COVID-19 and to provide narratives of how they felt about it at that time. This question was followed up by asking participants ‘Since that first time of hearing about the coronavirus/COVID-19, what has been the most helpful or useful sources of information for you to learn about the virus?’ and ‘What has made these sources so helpful or useful for you?’. It is on their responses to these three opening questions that we focus in this article.

## Results

### Initial reactions to COVID-19-related information

Given that initial news reports in Australian outlets focused on China and SARS [26], it is not surprising that most participants had first heard about the new infectious disease outbreak through news media sources reporting on the ‘mystery SARS-like’ cluster of cases in China. The location of the outbreak in first news reports and the comparison with SARS in this early news coverage led people to think that it was a faraway problem that would not directly affect them. Several people drew on their memories of previous outbreaks of novel infectious diseases such as SARS, MERS and Ebola in their responses. For example, Michael (aged 56) initially heard about the virus in Wuhan through television news reporting. He recalls hearing about coronavirus as it was being compared with SARS. He remembered that the SARS epidemic had not affected Australia, so did not think COVID-19 would either.

SARS seemed to affect other countries around the world but not Australia. So, I didn’t think that it would be as severe as what it ended up, so widespread across the world. So no, I didn’t really worry at the time.

Greg (aged 69) was even less concerned about the threat of COVID-19 at first, as his initial exposure to news about the coronavirus was via jokes that circulated on Facebook. He remembered that Facebook friends at first tended to make light of the threat of the outbreak: ‘I wasn’t too sure what to make of it, and enjoyed a couple of jokes when people said “I’m having a corona attack!” and put a photo of a [Corona brand] beer up on Facebook’. It was when Greg heard projections of the number of people that might be hospitalised with the virus in Australia on television news reporting in early March that he began to realise that it was a serious problem. The initial joking on Facebook was countered by the dramatic television news reports of the growing threat posed by COVID-19 to Australians.

I had listened to all the news broadcasts: listened with some trepidation to the forecasts of 'the hospitals are going to need thousands of beds', and concerned about that … By early March, I started to pay attention.

The initial sheer volume of news reporting and other public messaging about the spread of the coronavirus and measures needed to contain it could be overwhelming for some people. Because of the novel nature of the COVID-19 pandemic and the fast-changing news about it and its potential impacts on Australia, it was common for the participants to observe that they found themselves not being able to look away from news reporting about the crisis once the serious nature and rapid spread of the pandemic worldwide began to be reported in Australian news outlets. Participants commented on the importance of judicious consumption of news and information about the virus to avoid becoming overly obsessed and anxious after realising the risks to Australians of COVID-19. Several participants commented that they began to feel that there was saturation of ‘bad news’ and fear-inducing announcements from government officials in press conferences and health communication campaigns.

Some participants noted the tendency for sensationalism in news reporting and social media activity and the deleterious effects on their feelings of wellbeing. They often talked about ‘switching off’ from or limiting their exposure to news about the virus as time went on as a way of managing their distress and supporting their mental wellbeing. As Joe (aged 41) commented:

when I have looked at the international news and looking at what’s happening in America and that sort of stuff, it gets me really worked up and I get very upset about it. I find that quite challenging, and at the same time, I find it very difficult not to look. So, I found it really hard, particularly in the early days, in terms of just not constantly having the news on and constantly hearing about what was going on. It’s only probably been in the last two weeks that I’ve managed to sort of cut that down to maybe two or three times a week, whereas it was two or three times a day. It was just, I had to know what was going on all the time.

Several others reported difficulty in keeping up with all the new information being issued from these sources: some of which could be contradictory. For example, Emma (aged 29) described the government-provided information in press conferences or public health campaigns concerning restrictions as often ‘confusing’. She noted that some of the restrictions imposed by the government were ‘arbitrary’ or hard to make sense of. Emma gave the example of the number of people allowed at a wedding or a funeral, a rule which she remembered was constantly changed during the early months of the pandemic: ‘It’s, like, bizarre and kind of hard to understand’.

### Blame, misinformation and conspiracy theories

Many participants were highly aware of the potential for news reporting or social media content to be misleading or inaccurate: problems which themselves have received attention from the news media itself as well as public health authorities in Australia [5, 15]. Some people expressed feelings such as frustration, distress or anger around the kinds of information (conspiracies, misinformation, concerns about bias or fake news) circulating social media platforms. One example is Sarah (aged 54), whose husband is an essential worker in health services. She was concerned that the misinformation about COVID-19 in the news media and social media could reinforce or sanction careless or negligent behaviours that would place her husband and other frontline healthcare workers at increased risk of infection. She knew from her husband’s first-hand experience that the threat of COVID-19 was not exaggerated.

My husband was dealing with those patients who are highly contagious, and he was told not to wear full protection and that was frightening. So when people were saying ‘It’s a hoax, don’t worry about it,’ I’m like, ‘Well, you’re putting my husband’s life in danger’. So it was really distressing.

Other participants demonstrated a high level of scepticism towards the accuracy of information they encountered in the news media. They said that they were careful to try to evaluate the level of risk as it was reported in news outlets, given the news media’s tendency towards hyperbole to attract viewers. As James (aged 26) commented:

[The media] are going to catastrophise everything and anything, all the information. So, whatever I’m reading, I’ve got to make sure that I don’t just believe it straight away and look into it a bit more and ask some more questions, rather than just saying, yep, okay, I believe that.

For Greg (aged 69), the main source of information to learn about the novel coronavirus and COVID-19 has been television news reporting. He talked about the importance of ‘reading between the lines’ and being mindful of the polarisation and ‘bias of the media’ in reporting about the coronavirus. Greg was also concerned about some of the conspiracy theories that were circulating initially, and that the outbreak was not being taken seriously by a section of the community. He was even more vigilant in appraising the validity of news and commentary on Facebook: his second main source of information.

I must admit I’ve become quite careful about reading conspiracy-type theories on Facebook. Yeah, it’s a platform for everyone to have their say, but I’ve discovered that in my own opinion, some theories are quite farfetched. People can be sincerely wrong.

Joe (aged 41) said he is surprised by how many people he knows have ‘bought into’ the conspiracy theories, including his own elderly mother. He perceives these theories as expressed by people who need someone to blame. Joe said that he does his best to counter these claims where he has seen them expressed: which includes in face-to-face interactions or telephone conversations with close family members as well as in social media outlets such as Facebook.

There has been some discussion that I’ve had with family that has been just ridiculous. My mother, who’s a bit older and just, I don’t know, a bit susceptible to bad information, says all sorts of conspiracy-type things to me, which I’ve just told her is ridiculous. At one point, I think she was saying that China’s done this deliberately, and this was to break the world economy. I mean, the worst one I’ve seen and heard of, which was from an associate on Facebook, was the 5G theory, which – I just think – I don’t know … it seems to – a lot of people were buying into that, which surprised me. I think they were desperate to have a cause of something they could point at.

### Most trustworthy information sources

Given their caution about news reporting and social media content, many participants talked about being judicious around what sources of information they used to learn about the COVID-19 crisis. They placed an emphasis on trusted, unbiased, reliable sources of information that they assumed were founded on expert medical and scientific advice and research, or on personal experience of the pandemic.

Natalia (aged 67) was born overseas and keeps in close contact with friends and family there: including viewing content about COVID-19 they have shared on Facebook. She said that she is careful to check that any news items she sees her friends or family members sharing comes from ‘a well-known news source’ such as the ABC (Australian Broadcasting Corporation) or the *Washington Post* (USA) news outlet or quoting a scientific study: ‘I try to do that, because well, I know how fake news creates fear or hopes for nothing’. Ruth (aged 70) also referred to the ABC as well as the BBC (British Broadcasting Corporation) as trustworthy, noting that she uses her smartphone to access their news reporting.

I just keep reading on my phone and some articles I discount because I think they’re crap, and other articles I think, well, yeah, this seems to make sense … I take the ABC and the BBC as being okay.

For most participants, government sources such as the federal government health minister and state premiers and health authorities such as Chief Medical Officers were also viewed as credible. Greg (aged 69) said that he finds information from these sources to be the most helpful, mainly because he believes that ultimately, they have the country’s and its citizens’ best interests at heart. He positioned himself and other Australians as responsible for following government advice for the collective good of the community and as a way of demonstrating good citizenship.

Well that was pretty much the bottom line for me. That, okay, if the government says you’ve got to socially isolate, well that’s what I’ll do. I’ll take precautions, I’ll wear a mask, I’ll wear gloves when I go shopping. I did all of that in the early stages.

Max (aged 52) spoke about the value he placed on the federal government response communicated in regular news conferences that were closely covered by the news media. He liked keeping up to date with reporting of these news conferences because he thought that they provided the most current and local information about the pandemic and the current restrictions in a situation in which these details could change from day to day. Max found it reassuring and informative that these news conferences and announcements were predictable and appreciated being able to readily access these details using digital news outlets.

Even though those news conferences became a bit tedious and repetitive, it was good to know that they were regular conferences … and that you knew that a couple of times a day we were being updated as to what’s going on.

High value and trust were also placed on the information provided by people known personally to the participants who were considered to have expert knowledge or personal experience of the pandemic. Such sources included friends or family members who work in healthcare, government or science domains. They were viewed as unbiased and therefore more credible than some of the news media reporting. For example, Ruth (age 70) said that she trusts both her doctor and her brother, who is a scientist, to give her authoritative and fact-based advice about COVID-19.

I actually discussed it with my doctor, probably three or four weeks ago, because I see him frequently … He said in our particular district there hasn’t been any coronavirus cases for three or four weeks and he thought it was quite safe. So, I talked to him about it … I talk to my brother about it – he’s a scientist. I think it’s factual information and people with scientific backgrounds that provide the information.

Sarah (aged 54) noted that with her husband working in a hospital, their family had received a proliferation of COVID-19-related information from his workplace even before the national lockdown took place. She knew from her husband’s work experiences that hospitals were engaged in rushed preparations for a predicted surge of patients needing care for COVID-19: ‘Yeah that was the word of mouth we were getting. So that was, yeah, it was good in some respects and terrifying in others’.

Participants who had family members or friends living overseas also often nominated these people as important sources of details about what life was like in countries such as the USA, UK, Spain and Italy where the COVID-19 crisis was much further advanced than in Australia. Riley (aged 29), who was born in the USA and still has family and friends living there, observed that: ‘certainly once it hit New York, then I was getting inundated with messages from my parents, because it was affecting them very directly obviously’.

A small number of participants mentioned faith-based communities or teachings as contributing to their sense-making around COVID-19. For Greg (aged 69), it was his fundamentalist Christian teachings that contributed to his growing awareness that the COVID-19 outbreak in China could be serious globally, resulting in the ‘end times’ he believes is forecast in the Bible. For Riley (aged 29), the personal risk of infection was really brought home by new measures introduced into the synagogue that Riley regularly attends.

I was involved in a lot of stuff in the synagogue and about early to mid-March, early March, they were starting to say we can’t shake hands anymore and we can’t come close to each other anymore. When they started talking about that in the synagogue, I was starting to really pay attention, I was like ‘They’re telling me this for a reason!’. I started to take it a bit more seriously, so I’m glad that the people in my religious community were taking it seriously before I started to.

### Bringing sources together

As is evident from the participants’ accounts outlined above, many used a range of information sources about COVID-19. The relative influence of these sources in some cases changed as the pandemic gathered momentum or as key details about COVID-19 changed over the first 6 months when medical and public health experts were still learning about the ways the novel coronavirus spread, the effects of COVID-19 and how best to contain the pandemic and governments and health officials were struggling to find the most effective and least harmful policy settings.

Several people explained the complex processes by which they appraised and made sense of COVID-19 information through a range of sources. For example, Georgia (aged 24) commented that she likes the immediacy of sources of information like Twitter and television news reports but considers them not always trustworthy or reliable. Typically, she will supplement this information through her own online research using government websites and through word of mouth from friends who live overseas and have been more seriously affected or exposed to the COVID-19 crisis. Georgia explained that the government-sourced information is the most helpful for her because it is ‘verifiable’. She knows that the government draws on health expertise in formulating its COVID-19 advice and policy. In particular, she finds localised information most useful: for instance when and where it is safe to go outside in her local area, and what actions she should be taking to reduce her own risk of COVID-19 as well as risks to others. It is less important for her to learn about the ‘bigger picture’ of the pandemic. These practices also help Georgia deal with the plethora of information available about COVID-19: ‘Anything where the information is bite sized and verifiable, I appreciate, so I guess in that sense, Twitter is good as long as I then go fact check’.

Emma (aged 29) also receives a lot of news through Twitter, preferring to read a range of different sources on that platform so that she is then able to formulate her own views about the issue. She also recounted hearing in the news and social media about people’s real-life experiences of becoming ill with COVID-19 and how that was particularly powerful for her. Emma described herself as already living with anxiety pre-COVID-19. She noted that accessing more information and gaining knowledge about COVID-19 made her feel less worried: in part, because it gave her the knowledge to take precautions to avoid contracting the novel coronavirus. Emma was also keen to be aware of what the government was doing to handle the crisis, including how she as an individual could help the collective response.

Personally, I find it really helpful to have as much information as possible on things. I think that helps me relax a bit more. I know certain people, it’s the opposite, where the more you know about something, reading about something a lot, will make you more agitated. But it was the opposite for me – where I was like, I would like to know as much as possible about this so I can avoid it and knowing what the governments are doing and knowing what you can do personally to help and so on and so forth.

Another example of bringing different information sources together is provided by Darren (aged 64). He said that he has relied on government-related information in finding out and learning more about the coronavirus and COVID-19. He accesses this information via online government health websites. Darren commented that he finds this kind of information more truthful than the news media ‘spin’ that is imposed on government-based information.

I saw the media reports where health ministers and health advisors were giving information out, but to be quite honest I didn’t pay too much attention to it, because attached to all that was the media spin afterwards. So I left it alone to a great extent and just relied on the government website and blogs that were from medical personnel.

Darren noted that he is cautious about the circulation of ‘false information’ and ‘fake news’ on social media. However, he is willing to use social media to access websites and ‘serious’ bloggers which he accesses as more truthful and trustworthy: ‘They are either scientific or they are reliable blogs, if you know what I mean. They are ones that I have read for many, many years’.

The description of his evaluation of COVID-19 information sources provided by Mark (aged 48) highlights the importance of the advice offered both by international bodies that can provide general advice and local sources of information, as well as demonstrating that social media sites can be vital platforms for disseminating these details. Mark said that he has ‘never trusted the media for reporting anything’. He preferred the World Health Organization’s (WHO) regular media briefings hosted on social media outlets as his chief source of information about the novel coronavirus and COVID-19. Mark said that he used Australian government sources of information as a secondary source to the WHO, to provide more localised information and advice: for instance about guidelines and directives for daily living and how to prevent against contracting or spreading the coronavirus.

## Discussion

Similar to previous qualitative research on publics’ responses to information sources about new disease outbreaks [1, 2, 11, 36], our findings show that participants were active users of information sources rather than passively accepting news accounts, government spokespeople or social media content as authoritative. The participants demonstrated awareness that misinformation was rife in news outlets – and especially social media sites – and that they were taking steps to determine the accuracy of information. Their accounts also highlight the interactions of different forms of information sources, and the sophistication with which participants engage with these different kinds of information. Diverse sources of COVID-19-related information, both international and local, were actively accessed, appraised and engaged with by participants.

As was found in survey findings in Australia [35] and other countries [32], traditional media (television and radio news reports) were important sources for participants, as were government sources such as press conferences, health campaigns and websites [34, 35] and friends and family [34]. Despite contentions that Australian publics have lost confidence in the advice of public health authorities and governments due to conflicting and rapidly changing information provided [13], our participants demonstrated willingness to trust these sources for information and advice about how to respond to the crisis. Indeed, other research conducted around the same time as our study showed that Australians’ trust in government had increased dramatically since the outbreak of COVID-19: largely because they assess government interventions to manage COVID-19 as appropriate and effective [42]. Regular press conferences with government and health officials were important in gaining people’s trust and reassuring them that the federal and state governments were working hard to control the crisis. People wanted both very localised information that was directly relevant to them and general information from trusted global health organisations such as the WHO.

Healthcare professionals personally known to people, such as their regular general practitioner, were also trusted sources of information. Illustrative of the importance placed on experiential knowledge, the participants referred to the value of having informal discussions with friends and family members who were experts or working in relevant fields, such as healthcare or science, as well as engaging in-person in interactions with groups such as faith-based communities and simply hearing from friends and family who lived overseas about what COVID-19 conditions were like there.

Our findings support and extend other research that has highlighted the affective dimensions of engaging with information sources in relation to major health crisis such as outbreaks of new infectious diseases [2, 9–11]. Similar to survey-based research in Australia [35] and internationally [28–31, 33], our study’s participants reported a high interest in COVID-19 news reports in the initial stages of the pandemic. Some people described feelings of anxiety or distress in response to the plethora of information continually published in news reports and on social media. Others were angry and frustrated about the extent of misinformation that was circulating in the community and online and the potential for it to contribute to the spread of the coronavirus and pose a risk to others. However for many people, keeping up to date with changes in information and news in the rapidly changing environment of the COVID-19 crisis was a form of reassurance and helpful in ensuring they were conforming to best-practice risk avoidance and management.

The findings also show how certain key events or experiences made people become aware that the outbreak was threatening Australia and potentially themselves. For some people, this was hearing in the news media about the growing number of cases in their region, drastic government interventions imposed to contain the spread or the identification of infected people in their immediate locale. For others, it was face-to-face encounters or telephone conversations with trusted people or viewing content from friends and family members overseas on social media about how they were experiencing the pandemic in their countries that really brought home the dire threats posed by COVID-19 and what could happen to Australia if the outbreak were not contained.

A limitation of our study is that it did not involve a representative sample of Australian adults and therefore the findings are not generalisable to the population as a whole. However, a diverse group of participants was included, and the findings support and provide further detail about the trends identified in large-scale surveys of Australians’ news consumption and trust in information sources during the initial months of the COVID-19 crisis [34, 35].

## Conclusions

Our findings provide further contextual insights into the complexities and social contexts of these practices and sense-making responses, including how people bring together information from different sources in understanding the threat of COVID-19 and the interactions of digital with non-digital sources. A constantly changing news environment, as was the case during the first 6 months of the COVID-19 crisis, raises challenges for effective communication of risk and containment advice. People can become confused, distressed and overwhelmed by the plethora of information sources and fast-changing news environment. On the other hand, seeking out information can provide reassurance and comfort in response to anxiety and uncertainty. Clarity and consistency in risk messaging is important, as is responding quickly to changes in information and misinformation.

Our interview study took place at a certain point in the Australian experience of the COVID-19 crisis (towards the end of the national lockdown). Given the rapidly changing nature of the spread of COVID-19 in Australia since then, including a major outbreak in the state of Victoria and an extended second lockdown in that state, continuing and follow-up research is recommended to better understand how Australians have made sense of and protected themselves against the COVID-19 crisis and which sources have been most helpful for them in doing so.

## Data Availability

No data or materials are publicly available as the participants did not consent to open sharing of their interview transcripts or other personal information. Anonymised interview transcripts may be made available from the corresponding author on reasonable request.
